# Sex-Specific Differences in Patients with Sarcopenia and Peripheral Artery Disease

**DOI:** 10.3390/muscles5030063

**Published:** 2026-09-14

**Authors:** Volker H. Schmitt, Omar Hahad, Christoph Brochhausen, Thomas Münzel, Philipp Lurz, Christine Espinola-Klein, Lukas Hobohm, Karsten Keller

**Affiliations:** 1 Department of Cardiology, University Medical Center of the Johannes Gutenberg-University of Mainz, 55131 Mainz, Germany; mr.volkerschmitt@gmail.com (V.H.S.); omar.hahad@unimedizin-mainz.de (O.H.); tmuenzel@uni-mainz.de (T.M.); philipp.lurz@unimedizin-mainz.de (P.L.); espinola@uni-mainz.de (C.E.-K.); lukas.hobohm@unimedizin-mainz.de (L.H.); 2 German Center for Cardiovascular Research (DZHK), Partner Site Rhine Main, 55131 Mainz, Germany; 3 Institute of Pathology, Medical Faculty Mannheim, University Heidelberg, 68167 Mannheim, Germany; christoph.brochhausen-delius@umm.de; 4 Center for Thrombosis and Hemostasis (CTH), University Medical Center of the Johannes Gutenberg-University of Mainz, 55131 Mainz, Germany

**Keywords:** sarcopenia, peripheral artery disease, sex differences, clinical outcome, epidemiology, skeletal muscle impairment, mortality, epidemiology

## Abstract

**Background**: In patients with peripheral arterial disease (PAD), the presence of sarcopenia is associated with increased morbidity and mortality. However, sarcopenia is widely underrecognized and scientifically scarcely researched in PAD. Thus, we aimed to investigate sex-specific differences in PAD patients with sarcopenia. **Methods**: All hospitalizations due to PAD in Germany with additional coding for sarcopenia 2005–2020 were included in this analysis and stratified for patients’ sex. Female and male sarcopenic PAD patients were compared regarding patient characteristics, performed therapy and clinical outcome. **Results**: Overall, 3282 patient hospitalizations admitted due to PAD with sarcopenia were identified in Germany in 2005–2020. The majority of sarcopenic patient-cases were of male sex (N = 1821; 55.5%). Female PAD patients with sarcopenia were older (81.0 [75.0–87.0] vs. 76.0 [68.0–82.0] years, *p* < 0.001) and revealed a higher comorbidity burden mirrored by higher Charlson Comorbidity Index (7.0 [5.0–8.0] vs. 6.0 [3.0–8.0], *p* < 0.001). While surgical revascularization of the lower extremity was more often performed in hospitalizations of male sarcopenic PAD patients (17.0% vs. 14.0%, *p* = 0.018), interventional endovascular revascularization was similarly used in both sexes (12.7% vs. 11.8%, *p* = 0.429). Amputations were more often performed in male sarcopenic PAD patients (27.2% vs. 21.7%, *p* < 0.001). In-hospital death (8.9% vs. 6.5%, *p* = 0.009) and MACCE (11.0% vs. 9.1%, *p* = 0.070) occurred more often in female than in male sarcopenic PAD patients. **Conclusions**: We detected significant sex-specific differences in patient profile, performed treatment approaches, and mortality among hospitalized patients with PAD and concomitant sarcopenia. A low number of patients coded with sarcopenia indicates a remarkable under-recognition of sarcopenia in PAD.

## 1. Introduction

Peripheral artery disease (PAD) is a common and frequently detected atherosclerotic disease characterized by a reduced blood flow to the limbs due to narrowed arteries. PAD is crucially provoked by the classical cardiovascular risk factors including diabetes mellitus, smoking, arterial hypertension and hyperlipidemia, resulting in increased morbidity and mortality [1,2]. Sarcopenia leads to restricted physical performance due to a loss of muscle mass, a loss of muscle strength and a loss of muscle quality, and represents a widely under-recognized critical comorbidity in patients with PAD [1].

Muscle loss is a common state in PAD, decreasing quality of life and the prognosis of affected patients, especially in PAD patients with sarcopenia [1,3,4]. In critical limb ischemia, an additional sarcopenic condition was associated with elevated rates of cardiovascular events, but also amputation and mortality [5,6]. In daily clinical routine, sarcopenia is generally highly underrecognized by medical professionals and hence probably highly undiagnosed in patients with PAD [3]. Therefore, sarcopenia is a vastly underestimated threat in diseases like PAD [1].

Regarding PAD, differing clinical profiles of men and women were detected, including varying epidemiology, pathophysiology, risk factor burden, clinical presentation, performed therapeutic strategies, and outcomes including mortality [7]. Differences regarding the clinical manifestation of PAD between sexes may promote a delayed diagnosis in women. Women often present later during the disease progression and therefore show more advanced/aggravated disease stages at diagnosis, and may experience a higher rate of functional decline in combination with greater loss of mobility [8], leading to an increased loss of quality of life compared to men [9]. The presence of sarcopenia further complicates the clinical course of patients with cardiovascular diseases including PAD [1], leading to increased morbidity and mortality. Although sex differences in sarcopenia are barely assessed, various mechanisms seem to differ by sex. Women were shown to carry an approximate 20% increased risk of developing sarcopenia in comparison to men [10], and sex hormones seem to play a decisive role. Androgens and estrogens influence the metabolism of skeletal muscle and have an impact on the occurrence and progression of sarcopenia, hence representing possible and potential treatment strategies [11]. Despite an increasing awareness of sex-specific varieties, research on sarcopenia remains under-represented.

The primary objective of our study is to elucidate differences in clinical profile and outcome between male and female patients with PAD in co-prevalence with sarcopenia using data from German inpatient statistics based on ICD coding. Hence, in the present study, sex-specific differences in hospitalized PAD patients with sarcopenia were investigated.

## 2. Methods

### 2.1. Data Source

All patients hospitalized with a main diagnosis of coded PAD (ICD-code I70.2) and additional coding for sarcopenia (ICD-code M62.5) in Germany within the observational period including the years 2005 to 2020 were included (source: RDC of the Federal Statistical Office and the Statistical Offices of the federal states, DRG Statistics 2005–2020, and own calculations). The patients’ main diagnosis is defined as the diagnosis that was mainly and primarily responsible for the patients’ hospitalization (admission to the hospital) [12].

In Germany, the diagnoses of all hospitalized patients are coded and processed according to the diagnosis-related groups (DRG) system based on the established coding guidelines ICD-10-GM (International Classification of Diseases, 10th Revision with German Modification). Diagnostical, surgical and interventional procedures were acquired with OPS codes (surgery, diagnostic and procedures codes [Operationen- und Prozedurenschlüssel]) [1,13,14]. Data from all inpatient cases in Germany are gathered and processed by the Federal Statistical Office of Germany (Statistisches Bundesamt, Wiesbaden, Germany). The statistical analyses of the present study were undertaken on our behalf by the Research Data Center (RDC) of the Federal Bureau of Statistics (Wiesbaden, Germany); aggregated statistical results were provided by the RDC on the basis of our generated and supplied SPSS codes (IBM Corp. Released 2011. IBM SPSS Statistics for Windows, Version 20.0. IBM Corp.; Armonk, NY, USA).

In the present study, all hospitalization cases due to PAD (identified by the main ICD-code I70.2) with additional coding for sarcopenia (ICD-code M62.5) within an observational period of the years 2005 to 2020 in Germany were included. Cases were stratified regarding patients’ sex. Differences between men and women regarding patient profile, performed treatment strategies, amputation rate and adverse in-hospital events were assessed. Additionally, temporal trends were analyzed.

### 2.2. Definitions

Obesity was defined as a body mass index ≥ 30 kg/m^2^ according to WHO (World Health Organization) recommendations [14]. Shock and cardio-pulmonary resuscitation were defined according to current European guidelines [15,16]. Major amputations comprised surgeries with amputations above the ankle (OPS-code: 5-864), whereas minor amputations were defined as surgeries comprising amputations below the ankle (OPS-code: 5-865). Amputations of the upper extremities and amputations due to reasons other than limb ischemia, such as venous ulceration, trauma and malignancy, were consistently excluded from the analysis [17,18,19]. Surgical peripheral artery revascularization of the lower extremity included peripheral artery bypass operations, incision with embolectomy/thrombectomy and/or patch plastic operations of the legs (OPS codes 5-393.35, 5-393.36, 5-393.38, 5-393.42, 5-393.43, 5-393.44, 5-393.45, 5-393.46, 5-393.5, 5-393.6, 5-393.7, 5-380.7, 5-380.8, 5-395.7, 5-395.8), while peripheral endovascular intervention of the lower extremity comprised all interventional angioplasty treatments including balloon dilatation and stent implantation (OPS codes 8-836.0c, 8-836.0s, 8-836.1c, 8-836.1k, 8-836.2c, 8-836.2k, 8-836.3c, 8-836.3k, 8-840.0c, 8-840.1c, 8-840.2c, 8-840.3c, 8-840.4c, 8-840.5c, 8-840.0s, 8-840.1s, 8-840.2s, 8-840.3s, 8-840.4s, 8-840.5s) [1,14].

### 2.3. Outcome and Adverse In-Hospital Events

The primary study outcome was defined as in-hospital case fatality of all causes, whereas the secondary study outcome comprised major adverse cardiovascular and cerebrovascular events (MACCE, composite of all-cause in-hospital death, acute myocardial infarction [ICD-code I21], and/or ischemic stroke [ICD-code I63]). Additionally, the presence of further adverse in-hospital events like cardio-pulmonary resuscitation (CPR; OPS-code 8-77), pneumonia (ICD-codes J12–J18), pulmonary embolism (PE, ICD-code I26), deep venous thrombosis and/or thrombophlebitis (DVT, ICD-codes I80, I81, I82), myocardial infarction (MI, ICD-codes I21, I22), acute kidney injury (AKI, ICD-code N17), stroke (ischemic and hemorrhagic stroke, ICD-codes I61–64), intracerebral bleeding events (ICB, ICD-code I61), gastro-intestinal bleeding (GIB, ICD-codes K920–K922), and bleeding events with necessity of transfusion of blood components (OPS code 8-800) were analyzed.

### 2.4. Statistical Methods

Descriptive statistical comparisons were calculated as median and interquartile range (IQR) or as absolute numbers and corresponding percentages. Continuous variables were compared by using the Mann–Whitney-U test and for categorical variables using the Fisher’s exact or the chi^2^ test, as appropriate.

Temporal trends were calculated on an annual and age-dependent (age-decade) basis and presented descriptively as figures. In addition, we calculated linear regressions to investigate the increase in sarcopenia with patient age in male and female PAD patients, and an ANOVA to analyze the effect of age on the prevalence of sarcopenia in both sexes.

The investigation of the impact of sarcopenia on adverse in-hospital events and on in-hospital case fatality in female and male PAD patients was performed using univariable and multivariable logistic regression models given as odds ratio (OR) and 95% confidence intervals (CI). In addition, we analyzed the impact of patients’ sex on adverse in-hospital outcomes of PAD patients with sarcopenia. The multivariable regression models were adjusted for (I) adjustment with age, (II) age and Fontaine classification, (III) age, obesity, hyperlipidemia, cancer, coronary artery disease, heart failure, chronic obstructive pulmonary disease, essential arterial hypertension, acute and/or chronic kidney failure, diabetes mellitus and atrial fibrillation/flutter, as well as (IV) age, obesity, hyperlipidemia, cancer, coronary artery disease, heart failure, chronic obstructive pulmonary disease, essential arterial hypertension, acute and/or chronic kidney failure, diabetes mellitus, atrial fibrillation/flutter surgical, peripheral artery revascularization of the lower extremity and peripheral endovascular intervention of the lower extremity.

This epidemiological approach regarding the adjustment was used to guarantee the wide independence of the findings regarding the influence of sex on adverse in-hospital events during hospitalization [1,13,14]. Statistical significance was presupposed in cases of a *p*-value < 0.05 (two-sided). Statistical analyses were performed with the software SPSS^®^ (version 20.0; SPSS Inc., Chicago, IL, USA).

### 2.5. Ethical Aspects

In accordance with the German law, approval by an ethical committee and informed consent of the patients were not required, since the present study did not involve the direct accessing by us as the study investigators of the data of individual patients, but only on summarized/aggregated data provided by the RDC.

## 3. Results

**Figure 1:**
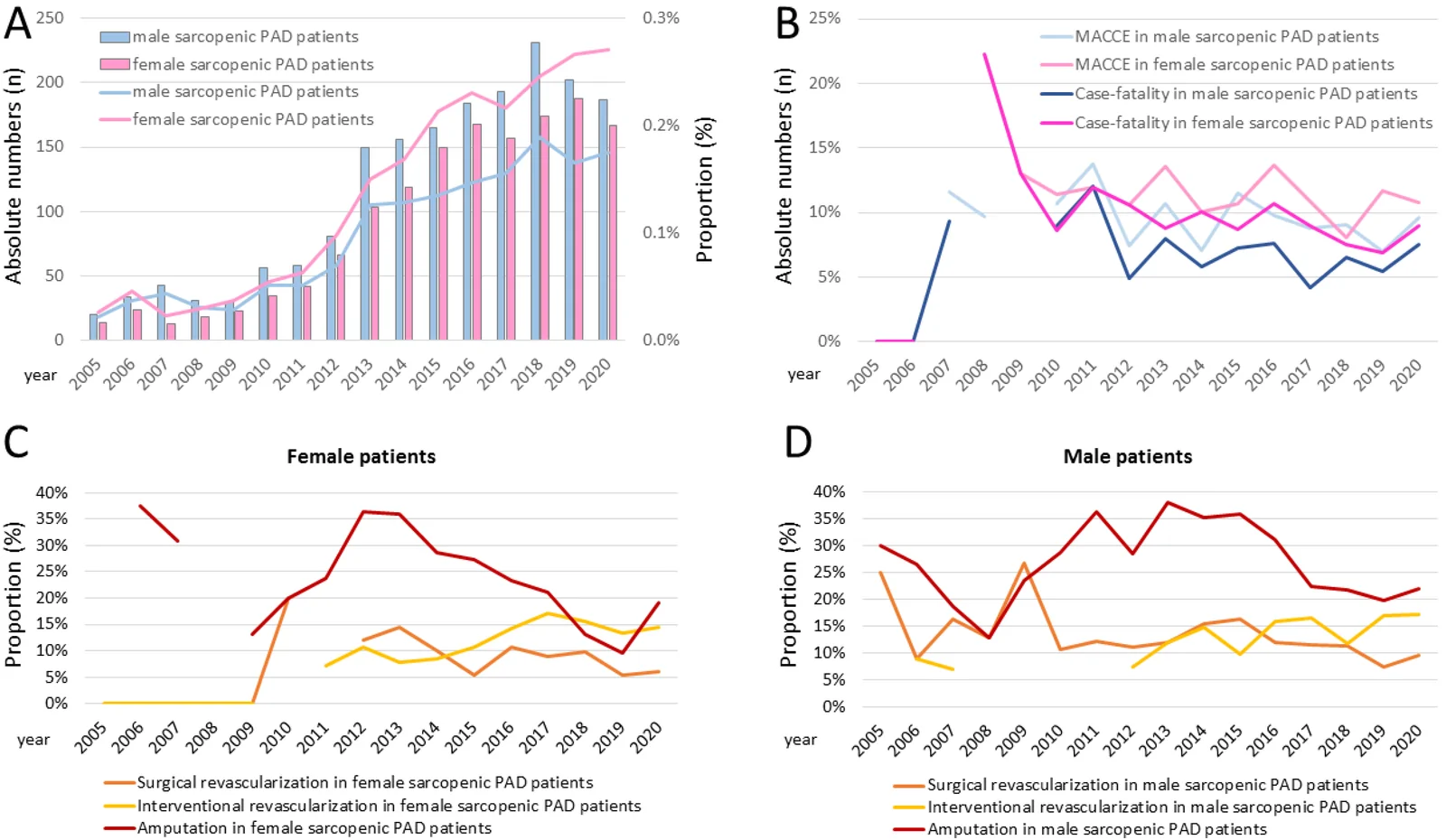
Temporal trends in hospitalized PAD patients with sarcopenia. (**A**) Annual trends of female and male PAD patient-cases with sarcopenia as well as in relation to all PAD patient-cases. (**B**) Annual trends of MACCE and case fatality in female and male PAD patient-cases with sarcopenia. (**C**) Annual trends regarding revascularization treatment and amputation in female PAD patient-cases with sarcopenia. (**D**) Annual trends regarding revascularization treatment and amputation in male PAD patient-cases with sarcopenia.

Overall, 2,825,635 PAD patient-cases (1,038,025 females and 1,787,610 males) were admitted to German hospitals during the observational period between 2005 and 2020. Among these, a small minority of 3282 (0.12%) hospitalization cases (1461 (0.14%) females and 1821 (0.10%) males) were additionally coded with the diagnosis of sarcopenia. The median age of all hospitalizations of PAD patients with additional sarcopenia was 78.0 (IQR 71.0–84.0) years, the median score of the Charlson Comorbidity Index was calculated as 6.0 (IQR 4.0–8.0) and the median length of in-hospital stay was 20.0 (IQR 12.0–30.0) days. Of the 3282 PAD patient-hospitalization cases with sarcopenia, 55.5% were males (N = 1821) and 44.5% were females (N = 1461; 44.5%) (Table 1). The annual total number of male sarcopenic PAD patients increased from 20 in the year 2005 to a peak of 231 in 2018, and decreased to 187 in 2020, whereas the annual absolute number of female sarcopenic PAD patients increased from 14 in 2005 to a peak of 188 in 2019, and was 167 in 2020. The calculated prevalence of sarcopenia in hospitalizations of PAD patients increased with age in both females (β 1.328 [1.132–1.524], *p* < 0.001) and males (β 1.258 [1.092–1.424], *p* < 0.001) in Germany. Age revealed a significant impact on the prevalence of sarcopenia in hospitalizations of PAD patients in the interaction ANOVA analysis, with a *p*-value of <0.001 in both sexes. The prevalence of sarcopenia in female PAD patients increased from 0.03% to 0.27%, while in males the rate increased from 0.02% to 0.18% (Figure 1).

**Figure 2:**
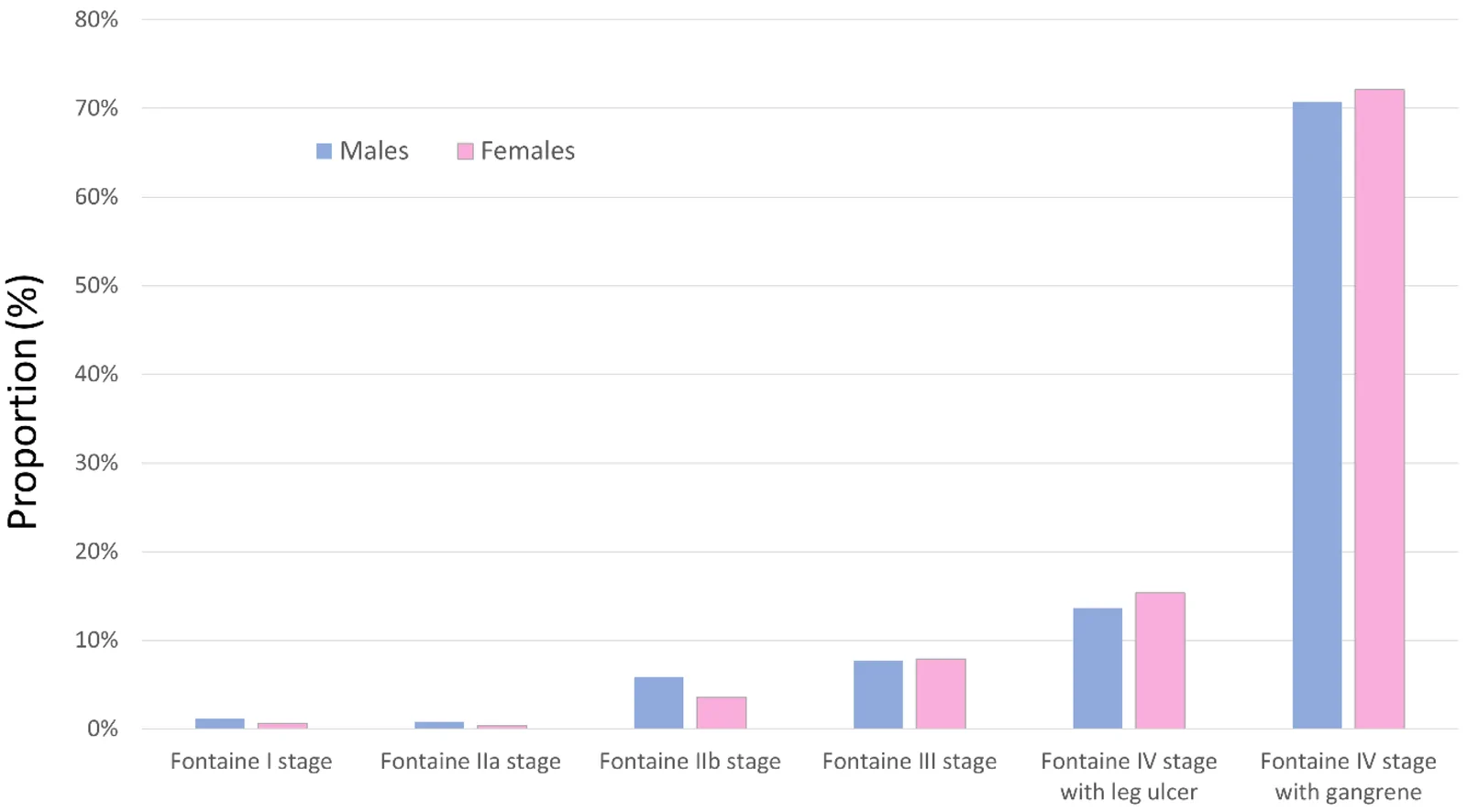
Categorization of sarcopenic PAD patients according to the Fontaine classification.

Regarding the severity of PAD categorized by the Fontaine classification, most female and male PAD patients admitted with sarcopenia were categorized into higher severity classes. In total, 84.4% of male and 87.5% of female sarcopenic PAD patient-cases revealed a PAD Fontaine stage IV (Figure 2).

**Figure 3:**
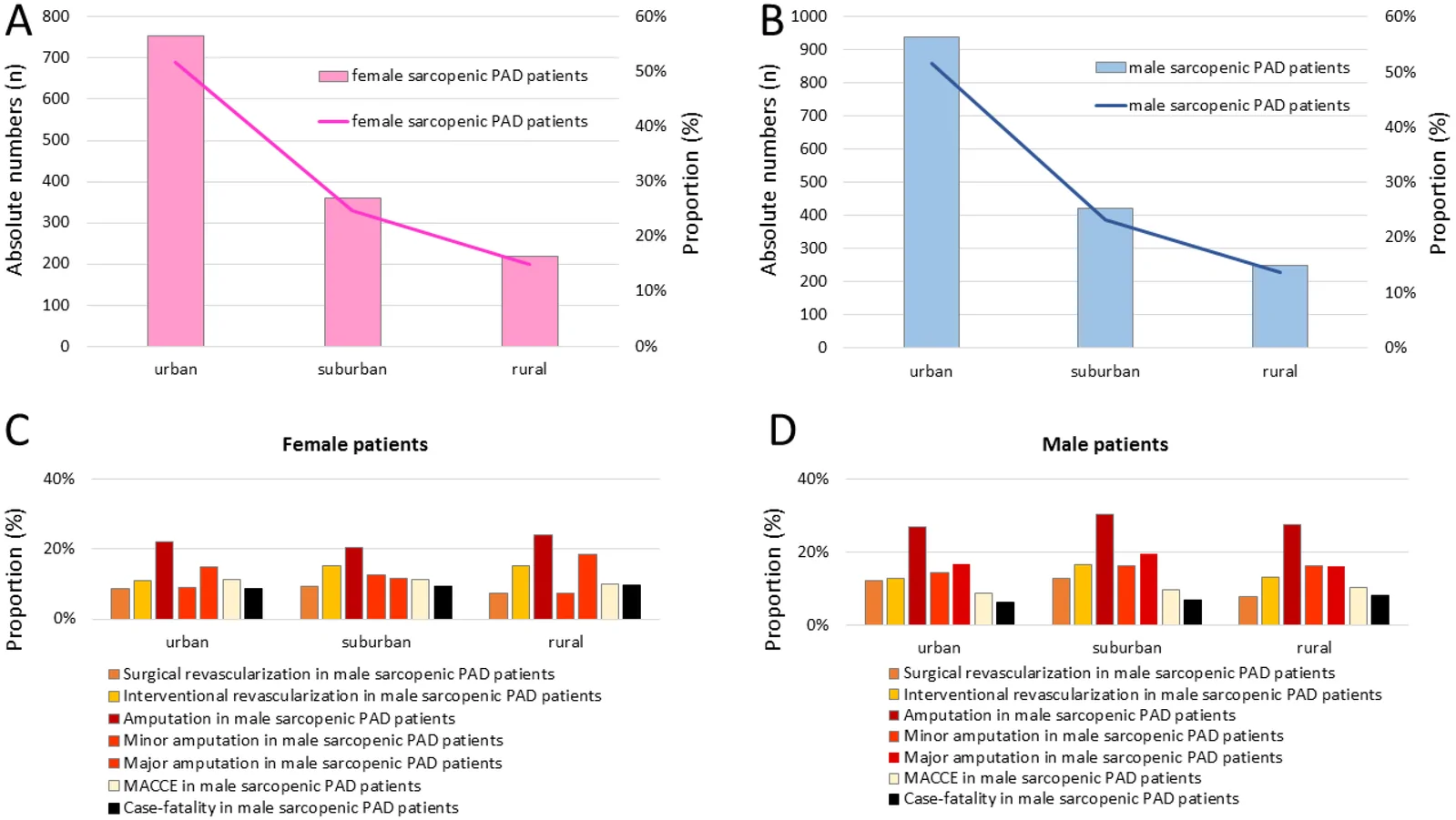
Regional trends in hospitalized PAD patients with sarcopenia. (**A**) Female PAD patients-cases with sarcopenia stratified by hospitals in urban, suburban and rural areas. (**B**) Male PAD patients-cases with sarcopenia stratified by hospitals in urban, suburban and rural areas. (**C**) Regional differences regarding revascularization treatment and amputation in female PAD patients. (**D**) Regional differences regarding revascularization treatment and amputation in male PAD patients.

The majority of sarcopenic female and male PAD patients were treated in urban hospitals, while the lowest rate was found in rural hospitals (Figure 3).

### 3.1. Patient Characteristics

Female PAD patients with sarcopenia were a median of 5 years older (81.0 [75.0–87.0] vs. 76.0 [68.0–82.0] years, *p* < 0.001) and revealed a higher comorbidity burden mirrored by a higher score of the Charlson Comorbidity Index (7.0 [5.0–8.0] vs. 6.0 [3.0–8.0], *p* < 0.001) (Table 1). Besides the diagnosis of diabetes mellitus, which was more frequently found in male PAD patients with sarcopenia (40.8% vs. 37.3%, *p* = 0.041), female and male sarcopenic PAD patients were comparable regarding the investigated cardiovascular risk factors profile. Female PAD patients with sarcopenia revealed higher rates of heart failure (37.1% vs. 32.2%, *p* = 0.003) and atrial fibrillation/flutter (32.6% vs. 29.4%, *p* = 0.048), whereas male patients had a higher prevalence of coronary artery disease (37.6% vs. 27.0%, *p* < 0.001). In addition, chronic obstructive pulmonary disease was more prevalent in the male sex (15.3% vs. 12.7%, *p* = 0.033), while acute or chronic kidney failure was more common in female sarcopenic PAD patients (48.9% vs. 45.2%, *p* = 0.033 (Table 1)).

### 3.2. Interventional and Surgical Treatments

In male PAD patients with sarcopenia, surgical revascularization of the lower extremities was more often performed compared to female sarcopenic PAD patients (17.0% vs. 14.0%, *p* = 0.018). Endovascular treatment of the lower extremities, in contrast, was similarly often applied in both sexes (12.7% vs. 11.8%, *p* = 0.429). Amputation rates were substantially higher in male compared to female sarcopenic PAD patients (27.2% vs. 21.7%, *p* < 0.001) (Table 1). Temporal trends regarding revascularization strategies and amputations in female and male PAD patients with sarcopenia are illustrated in Figure 1, whereas the regional differences are displayed in Figure 2.

Logistic regression analyses confirmed that revascularization by peripheral endovascular intervention in the lower extremity was less often performed in females (adjusted OR 0.459 [95%CI 0.391–0.538], *p* < 0.001), but also in males (adjusted OR 0.496 [95%CI 0.432–0.571], *p* < 0.001), with PAD and sarcopenia compared to PAD patients without sarcopenia. Also, surgical peripheral artery revascularization of the lower extremity was less widely applied in sarcopenic PAD patients (females: adjusted OR 0.763 [95%CI 0.658–0.885], *p* < 0.001; males: adjusted OR 0.830 [95%CI 0.734–0.938], *p* = 0.003). These results are independent of age, cardiovascular risk factors and comorbidities (Table 2).

While reperfusion treatments were less often applied in sarcopenic PAD patients, amputation was more often performed in female (adjusted OR 1.619 [95%CI 1.424–1.840], *p* < 0.001) and male (adjusted OR 1.830 [95%CI 1.643–2.040], *p* < 0.001) PAD patients with sarcopenia compared to PAD patients without sarcopenia (Table 2).

The decreased applications of peripheral endovascular intervention of the lower extremity (*p* = 0.479) and surgical peripheral artery revascularization of the lower extremity (*p* = 0.903) were not independent of age, cardiovascular risk factors and comorbidities (Table 3). In contrast, amputation (OR 1.284 [95%CI 1.083–1.522], *p* < 0.001) was independently associated with male sex in sarcopenic PAD patients. This was predominantly caused by an association between minor amputations and male sex, whereas no association was found regarding major amputations (Table 3).

### 3.3. Length of In-Hospital Stay

The length of in-hospital stay was similar in female and male PAD patients with sarcopenia (20.0 [12.0–29.0] vs. 20.0 [12.0–31.0] days, *p* = 0.509). Both sexes were related to a prolonged in-hospital stay of >10 days (females—OR 4.891 [95%CI 4.301–5.563], *p* < 0.001, males—OR 5.392 [95%CI 4.813–6.042], *p* < 0.001). No difference was present between female and male PAD patients with sarcopenia regarding a prolonged in-hospital stay >10 days (78.6% vs. 77.8%, *p* = 0.567) or >20 days (47.6% vs. 49.4%, *p* = 0.307) (Table 1). Overall, patients’ sex was not independently associated with a prolonged in-hospital stay in sarcopenic PAD patients (*p* = 0.940) (Table 3). This finding was consistent in all investigated age-decades (Table S3 of the Supplementary Materials).

### 3.4. Adverse In-Hospital Events

In-hospital mortality was significantly higher in female (+2.4%) compared to male sarcopenic PAD patients (8.9% vs. 6.5%, *p* = 0.009). Also, the occurrence of MACCE showed a trend towards significance, with higher rates in females than in males driven by short-term mortality (11.0% vs. 9.1%, *p* = 0.070). Accordingly, MACCE rate was significantly higher among women aged 60–69 years, whereas no significant elevation was present in all other age decades (Table S2 of the Supplementary Materials). No sex differences were present regarding all other adverse events (Table 1). Also, no age-dependant impact of sex on in-hospital death was found (Table S1 of the Supplementary Materials). The annual case fatality rate in female and male PAD patients with sarcopenia decreased during the observational period 2005–2020 (Figure 1). As illustrated in Figure 2, our data show that the in-hospital case fatality rate was lower in urban compared to rural hospitals.

The logistic regression analyses show an independent association between sarcopenia in PAD patients and an increased risk of in-hospital mortality in females (adjusted OR 1.378 [95%CI 1.141–1.666], *p* = 0.001), but not in male patients (adjusted OR 1.211 [95%CI 0.995–1.474], *p* = 0.056) (Table 2). Thus, the impact of sarcopenia in PAD patients is larger in females than in males. Sarcopenia was associated with an increased MACCE rate in both female (OR 1.417 [95%CI 1.193–1.684], *p* < 0.001) and male (OR 1.396 [95%CI 1.179–1.653], *p* < 0.001) PAD patients. While sarcopenia had no impact on the occurrence of myocardial infarction in both sexes, all other investigated adverse in-hospital events were more commonly detected in PAD patients with than without sarcopenia (Table 2).

Regarding the impact of patients’ sex on in-hospital mortality, a trend towards significance was present for females in terms of in-hospital death independent of age, cardiovascular risk factors and comorbidities (OR 1.287 [95%CI 0.978–1.694], *p* = 0.071) (Table 3, Figure S1 of the Supplementary Materials). In the age-dependent analysis, no association between female sex and in-hospital case-fatality was detected in any of the investigated age-decades (Table S1 of the Supplementary Materials). Female sex was independently related to a higher MACCE rate in the age group between 60 and 69 years (OR 1.634 [95%CI 1.006–2.655], *p* = 0.047) after adjustment of comorbidities and revascularization strategies, but not in any other age-decade (Table S2 Supplementary Materials). In addition, female sex was accompanied by a reduced risk of the development of pneumonia (OR 0.710 [95%CI 0.537–0.939], *p* = 0.017) in sarcopenic PAD patients (Table 3).

## 4. Discussion

In the present study, sex differences in hospitalized PAD patients with additional sarcopenia were investigated in real-world nationwide German inpatient statistics within a 16-year observational period (2005–2020). Sex-specific differences were observed regarding patient characteristics and profile, revascularization therapy performed, amputation rate, and survival. The main results could be condensed as follows: Hospitalized PAD patients with sarcopenia were more often of male than of female sex. Female PAD patients with sarcopenia were older, and revealed a higher comorbidity burden. In males, surgical treatments for revascularization of the lower extremity were more often performed compared to in females, whereas interventional endovascular procedures of the lower extremity were similarly used in both sexes. Amputations were more often required in male sarcopenic PAD patients. Sarcopenia was associated with increased mortality in both sexes. Although in-hospital mortality was higher in female compared to male sarcopenic PAD patients in the direct comparison of the groups, the logistic regression analyses did not demonstrate an independent association between female sex and an increased in-hospital mortality in sarcopenic PAD patients in any age-decade.

To our knowledge, this analysis represents the first assessment of the influence of sex on PAD patients with sarcopenia. PAD is a highly prevalent disease with more than 230 million suffering adults worldwide, accounting for 5.6% of the world population [20], whereas about 4–10% of adults aged 40 years or older are affected in the United States [7,21], and high hospitalization rates due to PAD are also identified in Germany [1]. Regarding this vast prevalence, the 3282 documented cases of patients hospitalized due to PAD with additionally coded sarcopenia within the investigated 16 years in Germany reflects and emphasizes the poor awareness of sarcopenia. These results show that sarcopenia is a highly underdiagnosed, overlooked and undertreated disease in PAD patients. It has to be assumed that most physicians who treat PAD patients think that (I) the patients’ muscle and strength loss is primarily a result of PAD as the underlying disease, and (II) the treatment of PAD is adequate to withstand the muscle and strength loss in this vulnerable patient group. This misleading assumption might be one key driver regarding the underdiagnosis and undertreatment of sarcopenia in this real-world sample of PAD patients in a well-developed industrial country with a well-equipped and functional healthcare system. In contrast to this misleading assumption, it is well known that, in sarcopenia occurring in patients with PAD, a treatment focussing only on PAD is not adequate to prevent (the aggravation of) sarcopenia. Our study results emphasize that sarcopenia is a driver of increased mortality in PAD patients. The in-hospital mortality was approximately 2.5% higher in female than in male PAD patients with coded sarcopenia. This finding underlines (I) the importance of detecting sarcopenia early in PAD patients and (II) the importance of not overlooking sarcopenia, particularly in women. Irrespective of PAD, sarcopenia is known to be highly underrecognized in daily clinical practice [22], and the awareness of sarcopenia is low in physicians other than geriatricians [23]. However, the small proportion of PAD cases with additional sarcopenia in the present study may also emphasizes the difficulties of distinguishing between PAD-related muscle loss and sarcopenia as an independent disease. Besides awareness of the disease, this might be the most important problem in the identification and coding of sarcopenia in PAD patients. The high proportion of PAD patients suffering from Fontaine stages III and IV underlines that the majority of cases who were admitted to hospital had an end-stage peripheral artery disease with acute or chronic limb ischemia (Figure 2). Studies have shown that PAD severity bares a significant relation to elevated muscle mass loss in the lower limbs, and therefore muscle loss could primarily be assigned to the underlying disease and not to additional sarcopenia [24].

In general, sarcopenia is highly prevalent in PAD patients, which was impressively demonstrated by studies such as the Korea National Health and Nutrition Survey [25]. This assessment comprised more than 14,000 adults who underwent sarcopenia diagnostic test and health surveys, in which sarcopenia was detected in 31.3% of all participants. Regarding sex, the prevalence of sarcopenia was double in females (40.4%) compared to males (20.2%). In line with this, results from the English Longitudinal Study of Ageing, an observational study from the United Kingdom on the elderly, revealed a 14.5% prevalence of sarcopenia in this population and a 20% higher risk in women of developing sarcopenia compared to men within an 8-years follow-up period [10]. These impressive data underscore the underdiagnosis of sarcopenia in the present study, and generally in daily clinical routine, since ICD coding mirrors the daily practice of diagnosing in hospitals. In daily clinical routine, special diagnostics regarding sarcopenia are often lacking due to several reasons; besides the poor level of recognition and the underestimation of the disease, the complexity of the diagnostic approaches to sarcopenia is also a common issue [26,27]. Also, no routine screening for sarcopenia exists in German hospitals. Importantly, it has to be remembered that the present study exclusively relied on ICD coding, and the diagnosis was hence not based on current consensus definitions (e.g., EWGSOP2), which include muscle strength and physical performance. The low case number of PAD patients with coded sarcopenia in our present study is likely driven by the underdiagnosis of sarcopenia. Consequently, the majority of men detected with PAD and sarcopenia may also represent a diagnostic bias related to underdiagnosing sarcopenia in the majority of affected PAD patients. In addition, it is well known that patients with (chronic and especially chronically progressive) cardiovascular diseases including PAD often suffer from chronic comorbidities like diabetes mellitus or chronic obstructive pulmonary disease, which hamper patients’ exercise capacities, decrease their quality of life, and are accompanied by a body-wasting process with muscle mass loss [28]. This general concept of body-wasting is applied in all diseases that are related to losses of tissue and an induced primary catabolic state, but are more precisely termed cachexia with/and/or sarcopenia [28]. Indeed, PAD as a chronic condition is an important reason for the development of sarcopenia [28]. Yet, since the sarcopenic state might be interpreted as one aspect of an end stage of PAD, and not recognized as an additional disease by healthcare professionals, sarcopenia will not be recorded, leading to low coding frequency and a significant under-coding.

The aforementioned studies from Korea and the United Kingdom revealed a varying prevalence regarding sarcopenia between females and males, as well as several sex-specific differences in sarcopenia. In the first study from Korea [25], the prevalence of sarcopenia was doubled in females (40.4%) compared to males (20.2%). In line with this, in the second study in the United Kingdom, a 20% higher risk of developing sarcopenia was found in women in comparison to men within an 8-year follow-up period [10]. In our present study assessing real-world nationwide German inpatient statistics, more male than female patients with PAD and sarcopenia were detected and coded. However, this result must be handled with caution because of the lower prevalence rates of sarcopenia than expected, and this may be due to the under-recognition of sarcopenia in the present study. According to results from the Global Burden of Disease Study, females seem to outnumber males regarding prevalence of PAD in all age groups [22,29]. In both studies the Korean and the study with its origin in the United Kingdom, patients were explicatively examined for the presence sarcopenia, whereas our study elucidates real-world data from daily clinical practice, without strict assessments of sarcopenia. It can be assumed that the under-recognition of sarcopenia is a general problem similar to in many other countries. Several reasons are conceivable for this under-recognition of sarcopenia, including the specialized and complex testing process that is rarely performed in the daily routines of PAD patients’ management in hospitals, which are time-consuming and require special examinations to identify sarcopenia. For both, there is often no time or room in the daily clinical routine, especially when there is a lack of understanding regarding the importance of this disorder/deviation to patient outcomes. Consensus definitions like EWGSOP2 have been available for a couple of years, but are a long way from being included in the daily practice for treating PAD patients. However, even these more modern methods remain complex and demand resources in an increasingly narrowing healthcare system. The impact of sarcopenia on PAD patients‘ outcomes shown in our study underlines the need for further improvements in diagnostic tools so as to simplify the processes of screening and diagnosing sarcopenia, and to ensure a greater awareness and implementation of screening tools in the daily routine for female and male PAD patients.

Regarding risk factors, in the study from Korea, in men, the risk of sarcopenia was associated with older age and life without a spouse, whereas the risk of women was increased by high-density lipoprotein cholesterol, alcohol consumption and aerobic exercise. In both sexes, a low body mass index was related to an increased risk for sarcopenia, as was no and, surprisingly, even moderate resistance exercise [25]. In this context, it has to be taken into account that ethnic, dietary, socioeconomic, and healthcare system differences may limit the comparability of the results between Germany and Asia. Sarcopenia is known to be associated with metabolic syndrome, and both interact with and partly reinforce each other. Metabolic syndrome is caused by cardiovascular risk factors like arterial hypertension, diabetes mellitus, hyperlipoproteinemia and obesity, which in turn also promote atherosclerotic diseases including PAD [3,19,30]. Importantly, sarcopenia is often overlooked, and therefore underestimated, in patients with obesity [3,31]. Regarding sex differences, only a few studies exist, and these came to inconsistent findings; while some studies demonstrated a stronger association between sarcopenia and the metabolic syndrome in females compared to males [32], a Japanese study found no correlations between the metabolic syndrome and sarcopenia, but abdominal obesity was found to be a main contributor to the association between metabolic syndrome and sarcopenia across both sexes [33]. In line with these findings, female and male PAD patients carry differing risk profiles for PAD; smoking and history of stroke and/or myocardial infarction are more strongly associated with the risk of PAD in women compared to men, whereas higher levels of high-density lipoprotein cholesterol are identified to be more beneficial in women [34]. Sex differences in PAD have multiple explanatory factors, such as differing risk factor burdens (including lipid profiles, plaque morphologies, immune and inflammatory responses), hormonal disparities, and differing responses to vascular stressors [7,35]. In addition, women have smaller vessel diameters than males [36]. In the present study, females revealed a higher prevalence of cardiovascular risk factors in combination with a worse comorbidity burden compared to males. For patients, prevention and, at manifestation of PAD, best possible treatment are crucial to avoiding PAD disease and its aggravation. In this regard, an interesting recently published study from Park et al. assessed the impacts of individual changes in body composition on the development of cardiometabolic disease in men and women with and without sarcopenia [37]. While a decrease in obesity and gain of muscle were beneficial in men without sarcopenia, an inverse relationship between weight and cardiometabolic diseases was found in women without sarcopenia. In the presence of sarcopenia, no cardiometabolic benefit of increasing lean mass was detected regardless of sex. Diabetes mellitus was highly prevalent in the cohort of the present study (40.8% in men, 37.3% in women), mirroring the relevance of the metabolic syndrome and insulin resistance seen in PAD and progressive muscle mass loss to sarcopenia. Insulin resistance and advances in glycation end-products in diabetic patients cause inflammation and oxidative stress, which in turn cause atherosclerosis, vascular damage, and cellular as well as mitochondrial dysfunction, and may lead to cell death [38,39,40]. This state promotes skeletal muscle loss, fostering the development of sarcopenia. On the other hand, since skeletal muscle is crucial to glucose uptake [40], reduced muscle mass can affect glucose metabolization, resulting in an enhanced risk of diabetes mellitus in sarcopenic patients [39]. Inflammation and oxidative stress also represent relevant underlying pathomechanisms for the genesis of atherosclerosic diseases including PAD. Reactive oxygen species provoke endothelial as well as mitochondrial dysfunction. In PAD, reactive oxygen species are often elevated due to inflammation and platelet activation, both of which promote the further endothelial-derived generation of reactive oxygen species, which again results in even greater platelet activation, leading to a vicious cycle of reactive oxygen species formation. As a result, oxidative stress and inflammation provoke endothelial dysfunction and atherosclerosis [1,41,42].

The effects of sex hormones are important reasons for sex disparities in sarcopenia. The anabolic effects of androgens on skeletal muscle are well-known, whereas the effect of estrogen on skeletal muscle remains controversial up to now. Nevertheless, sex hormones seem to play an important role in the pathophysiology of sarcopenia, and therapeutic androgen replacement has shown positive effects for treating sarcopenia, but potential side effects do exist [11,43,44].

In PAD, functional decline was shown to occur earlier and faster in women than in men, leading to decreased walking distances and walking speeds as well as disabled mobility during a 4-year follow-up period, which was associated with a smaller baseline calf muscle area in women [8]. Aiming for the best possible maintenance of health and quality of life in both PAD and sarcopenia, physical training is essential [45,46]. Since loss of muscle mass is one key ingredient of sarcopenia, resistance training is crucial to improving skeletal muscle mass and muscle function. However, the typical claudication pain in PAD might hamper this training [47]. Regarding sex differences in muscle metabolism, resistance training is especially important in women [46].

Additionally, the revascularization of occluded arteries in patients with PAD also has beneficial effects on sarcopenia, as shown by the muscle growth in the lower leg alongside an increased post-interventional ankle-brachial index, whereas in patients without post-interventional improvement of the ankle-brachial index a further decline of the musculature of the lower legs was found [48]. Although the proportion of male PAD patients with a PAD Fontaine stage IV exceeds the proportion of female PAD patients slightly, both surgical revascularization and amputations were more frequently performed in men. This finding of a lower usage of revascularization strategies was driven by older age and comorbidities, which was demonstrated in the logistic regressions, showing that female sex was associated—not independently of age and comorbidities—with a lower usage of revascularization strategies. Thus, the lower revascularization rates in female patients might be triggered by clinical judgment (given their older age and higher comorbidity burden). However, causality cannot be inferred from the available data, and hence it remains unclear whether these results reflect therapeutic decision-making, increased frailty, anatomical disease complexity or treatment limitations due to comorbidity, or are caused by the coding bias that these data unfortunately contain.

Revascularization in PAD leads to an improved mitochondrial activity of the calf muscle, as well as increased myofiber health and capillary density with simultaneously increased walking distance [49]. Therefore, patients with PAD and sarcopenia require the best possible treatment of cardiovascular risk factors, physical training with a special focus on resistance training in regard to sarcopenia, as well as reperfusion strategies if indicated to maintain or regain mobility.

Our study revealed a substantially higher rate of in-hospital mortality in female compared to male sarcopenic PAD patients in the direct comparison of the groups. However, this finding was not confirmed either in the adjusted logistic regressions or in the age-dependent analysis regarding the impact of female sex on in-hospital mortality in the different age-decades. Thus, the higher in-hospital mortality of female PAD patients with sarcopenia was primarily driven by an aggravated comorbidity profile. Sarcopenic patients with PAD represent a highly vulnerable but widely unrecognized patient group in which sex plays a decisive role regarding disease development and clinical course, whereas little is as yet known about sex-specific pathophysiology and mechanisms. Improvements in awareness and routinely performed diagnostics are essential first steps, and further research on this issue is also essential to developing adjusted therapeutic strategies.

## 5. Limitations

Several important limitations should be mentioned regarding the present study. Generally, due to the nature of an ICD- and OPS code-based study on hospitalized patients, under-reporting and under-coding represent a possible bias. Also, data are only available for the time of the in-hospital stay, and hence no follow-up assessment is possible due to the limited time-frame. Furthermore, no data are available on concomitant medication or laboratory markers (e.g., HbA1c and lipid profiles for evaluating metabolic health). Also, repeated hospitalizations of the same patient could have been included in the database, as DRG data are generally case-based rather than patient-based.

Regarding the present study, the low number of patients coded with sarcopenia in particular demonstrates on the one hand the low awareness—resulting in the vast under-recognition and under-coding—of sarcopenia in PAD patients in Germany within the investigated timeframe. In ICD coding practice, sarcopenia is systematically underdiagnosed and undercoded particularly in non-geriatric settings, since muscle loss may be partly attributable to the underlying disease. It cannot be ruled out that this might also influence the results of the sex-specific comparisons in the present study. On the other hand, this small proportion of PAD cases with sarcopenia underlines the difficulties of distinguishing between PAD-related muscle loss and sarcopenia as an independent disease. This might be the most important problem in the identification and coding of sarcopenia in PAD patients. Although differential coding rates of sarcopenia between men and women are possible, a systematic undercoding concerning one sex more than the other is hard to prove. Limitations related to coding failure are within the nature of ICD-coded studies, and therefore represent a possible bias of this study type; nevertheless, in the case of sarcopenia as a generally vastly underdiagnosed disease, a sex-dependent bias could not be ruled out. Furthermore, in the present study, sarcopenia was exclusively identified through the ICD-10-GM code M62.5, yet modern consensus definitions like EWGSOP2 and AWGS 2019 require the integration of muscle mass, muscle strength, and physical performance measurements. It has to be considered that administrative coding-based sarcopenia identification captures only a small and likely atypical subset of true sarcopenia cases, and the clinical threshold for assigning the ICD code of sarcopenia may systematically differ across hospital types, age groups, and sexes. On the other hand, the present dataset represents patients in which sarcopenia and PAD were confidenlty and validly identified within a large timeframe in Germany, and therefore provides a precious patient population whose investigation may help to elucidate patient characteritics and clinical differences, such as sex-specific patterns. Sarcopenia is a highly underrecognized condition that is challenging to diagnose in clinical practice, and has fortunately attarcted gretaer attention in the last few years, including seeing the development of modern diagnostic tools and definitions. These were not available during the entire investigation period of the present study. But even if tools were available within the complete timeframe, due to the nature of ICD-coded studies, no data regarding the diagnosis method would be withdrawable. In sarcopenia, there were no standardized diagnostic criteria for the coding of M62.5 across hospitals, especially within the investigated time period, leading to possible inter-hospital variations in diagnostic approaches. In addition, due to the ICD-based assessment approach, no data are available to further evaluate the ankle-brachial index, which should be mentioned as a main limitation of the study. Hence, findings such as the differences in surgical revascularization and amputation rates, which were less frequently performed in women in the present study, may be related to appropriate clinical judgments (older age, higher comorbidity burden), systemic treatment biases, or disease severity at presentation. Undoubtedly, further studies are necessary to further investigate sarcopania in PAD patients, including in making sex-specific distinctions, especially as regards the beforementioned limitations. Nevertheless, the present study includes a considerable population of PAD patients with secured sarcopenia, and may help to provide some more insights into this highly underrepresented scientific issue.

## 6. Conclusions

Sarcopenia in general represents a decisive risk factor in patients with PAD, which is to this day highly underestimated and widely unconsidered in daily clinical life. The present study indicates differences between men and women who were hospitalized due to PAD and also had sarcopenia regarding patient characteristics, risk factor and comorbidity profiles, performed therapy approaches, as well as in-hospital adverse events and mortality. Also, a vast undercoding of sarcopenia was seen, reflecting the huge under-recognition of this disease in patients with PAD. These results clearly demonstrate the urgent need for further research on sarcopenia in patients with PAD to improve the recognition, diagnosis, and clinical outcome of this vulnerable patient group. Research on varying pathomechanisms of sarcopenia between men and women might one day lead to more individualized treatment strategies. However, as a first step, the awareness and recognition of sarcopenia has to be improved in daily clinical routine in order to at least identify affected patients, particularly in special patient groups such as people with PAD.

## Figures and Tables

**Table 1:** Patient characteristics, medical history, presentation and adverse in-hospital events for the 3282 hospitalizations of patients admitted due to PAD with coded sarcopenia in Germany in the years 2005–2020, stratified for patients’ sex. Bold indicates significant differences at *p* ≤ 0.05.

Parameters	Male PAD Patients with Sarcopenia (N = 1821; 55.5%)	Female PAD Patients with Sarcopenia (N = 1461; 44.5%)	*p*-Value
Age	76.0 (68.0–82.0)	81.0 (75.0–87.0)	**<0.001**
In-hospital stay (days)	20.0 (12.0–31.0)	20.0 (12.0–29.0)	0.509
In-hospital stay > 10 days	1417 (77.8%)	1149 (78.6%)	0.567
In-hospital stay > 20 days	903 (49.4%)	698 (47.6%)	0.307
Cardiovascular risk factors			
Obesity	148 (8.1%)	126 (8.6%)	0.609
Essential arterial hypertension	1041 (57.2%)	866 (59.3%)	0.224
Diabetes mellitus	743 (40.8%)	545 (37.3%)	**0.041**
Hyperlipidaemia	581 (31.9%)	434 (29.7%)	0.175
Comorbidities			
Heart failure	586 (32.2%)	542 (37.1%)	**0.003**
Coronary artery disease	684 (37.6%)	395 (27.0%)	**<0.001**
Atrial fibrillation/flutter	535 (29.4%)	476 (32.6%)	**0.048**
Chronic obstructive pulmonary disease	278 (15.3%)	185 (12.7%)	**0.033**
Acute or chronic kidney failure	823 (45.2%)	715 (48.9%)	**0.033**
Cancer	72 (4.0%)	52 (3.6%)	0.556
Chronic anaemia	391 (21.5%)	307 (21.0%)	0.750
Charlson comorbidity index	6.0 (3.0–8.0)	7.0 (5.0–8.0)	**<0.001**
Treatment			
Amputation	496 (27.2%)	317 (21.7%)	**<0.001**
Minor amputation	266 (14.6%)	136 (9.3%)	**<0.001**
Major amputation	307 (16.9%)	214 (14.6%)	0.085
Peripheral endovascular intervention of the lower extremity	231 (12.7%)	172 (11.8%)	0.429
Surgical peripheral artery revascularization of the lower extremity	309 (17.0%)	204 (14.0%)	**0.018**
Intensive care unit	250 (13.7%)	159 (10.9%)	**0.014**
Adverse events during hospitalization			
In-hospital case fatality	118 (6.5%)	130 (8.9%)	**0.009**
MACCE	166 (9.1%)	161 (11.0%)	0.070
Cardio-pulmonary resuscitation	38 (2.1%)	28 (1.9%)	0.730
Myocardial infarction	32 (1.8%)	22 (1.5%)	0.574
Shock	43 (2.4%)	39 (2.7%)	0.574
Deep venous thrombosis and/or thrombophlebitis	25 (1.4%)	23 (1.6%)	0.633
Pulmonary embolism	8 (0.4%)	8 (0.5%)	0.802
Acute kidney failure	161 (8.8%)	127 (8.7%)	0.881
Pneumonia	150 (8.2%)	94 (6.4%)	**0.050**
Stroke (ischemic or hemorrhagic)	29 (1.6%)	20 (1.4%)	0.600
Gastro-intestinal bleeding	35 (1.9%)	31 (2.1%)	0.685
Transfusion of blood constituents	481 (26.4%)	377 (25.8%)	0.693

**Table 2:** Impact of sarcopenia on in-hospital case fatality and adverse events during in-hospital stay in male and female patients admitted due to PAD of any age (univariate and multivariate logistic regression model). Bold indicates significant differences at *p* ≤ 0.05.

	Male Patients								Female Patients							
	Univariate Regression Model		Multivariate Regression Model Adjusted or Age		Multivariate Regression Model Adjusted for Age and Fontaine Classification		Multivariate Regression Model *		Univariate Regression Model		Multivariate Regression Model Adjusted or Age		Multivariate Regression Model Adjusted for Age and Fontaine Classification		Multivariate Regression Model *	
	OR (95% CI)	*p*-Value	OR (95% CI)	*p*-Value	OR (95% CI)	*p*-Value	OR (95% CI)	*p*-Value	OR (95% CI)	*p*-Value	OR (95% CI)	*p*-Value	OR (95% CI)	*p*-Value	OR (95% CI)	*p*-Value
**Adverse events during in-hospital stay**																
In-hospital case-fatality	2.695 (2.236–3.249)	**<0.001**	1.884 (1.561–2.274)	**<0.001**	1.184 (0.981–1.428)	0.079	1.211 (0.995–1.474)	0.056	2.675 (2.234–3.204)	**<0.001**	2.052 (1.709–2.464)	**<0.001**	1.398 (1.164–1.679)	**<0.001**	1.378 (1.141–1.666)	**0.001**
MACCE	2.884 (2.458–3.383)	**<0.001**	2.128 (1.811–2.500)	**<0.001**	1.379 (1.174–1.621)	**<0.001**	1.396 (1.179–1.653)	**<0.001**	2.671 (2.267–3.147)	**<0.001**	2.114 (1.790–2.496)	**<0.001**	1.471 (1.246–1.737)	**<0.001**	1.417 (1.193–1.684)	**<0.001**
In-hospital stay > 10 days	7.091 (6.348–7.920)	**<0.001**	6.359 (5.690–7.106)	**<0.001**	4.062 (3.608–4.573)	**<0.001**	5.392 (4.813–6.042)	**<0.001**	6.642 (5.861–7.528)	**<0.001**	5.979 (5.270–6.782)	**<0.001**	3.927 (3.446–4.475)	**<0.001**	4.891 (4.301–5.563)	**<0.001**
In-hospital stay > 20 days	6.258 (5.709–6.859)	**<0.001**	5.551 (5.060–6.089)	**<0.001**	3.261 (2.956–3.597)	**<0.001**			5.294 (4.778–5.866)	**<0.001**	4.785 (4.315–5.307)	**<0.001**	3.022 (2.716–3.363)	**<0.001**		**<0.001**
Pneumonia	4.603 (3.894–5.442)	**<0.001**	3.580 (3.029–4.231)	**<0.001**	2.333 (1.973–2.758)	**<0.001**	2.336 (1.960–2.784)	**<0.001**	4.166 (3.382–5.132)	**<0.001**	3.516 (2.852–4.335)	**<0.001**	2.410 (1.954–2.972)	**<0.001**	2.172 (1.752–2.694)	**<0.001**
Deep venous thrombosis and/or thrombophlebitis	3.074 (2.071–4.565)	**<0.001**	2.901 (1.953–4.308)	**<0.001**	2.300 (1.548–3.417)	**<0.001**	2.481 (1.669–3.689)	**<0.001**	2.530 (1.675–3.822)	**<0.001**	2.325 (1.539–3.514)	**<0.001**	1.993 (1.318–3.012)	**0.001**	1.924 (1.272–2.910)	**0.002**
Pulmonary embolism	4.110 (2.050–8.244)	**<0.001**	3.664 (1.826–7.351)	**<0.001**	2.555 (1.273–5.128)	**0.008**	2.459 (1.223–4.944)	**0.012**	3.835 (1.911–7.698)	**<0.001**	3.596 (1.791–7.220)	**<0.001**	2.609 (1.299–5.241)	**0.007**	2.360 (1.173–4.750)	**0.016**
Acute renal failure	5.136 (4.367–6.040)	**<0.001**	3.839 (3.258–4.524)	**<0.001**	2.456 (2.084–2.894)	**<0.001**	2.445 (2.050–2.917)	**<0.001**	4.414 (3.682–5.290)	**<0.001**	3.682 (3.068–4.418)	**<0.001**	2.439 (2.032–2.928)	**<0.001**	2.142 (1.764–2.601)	**<0.001**
Cardio-pulmonary resuscitation	3.266 (2.368–4.507)	**<0.001**	2.731 (1.987–3.755)	**<0.001**	1.767 (1.285–2.430)	**<0.001**	1.663 (1.199–2.305)	**0.002**	3.224 (2.216–4.690)	**<0.001**	2.966 (2.039–4.316)	**<0.001**	2.016 (1.385–2.934)	**<0.001**	1.813 (1.241–2.649)	**0.002**
Myocardial infarction	2.081 (1.466–2.953)	**<0.001**	1.739 (1.231–2.456)	**0.002**	1.251 (0.886–1.767)	0.204	1.089 (0.763–1.555)	0.637	1.778 (1.167–2.711)	**0.007**	1.567 (1.028–2.389)	**0.037**	1.192 (0.781–1.817)	0.416	0.995 (0.648–1.527)	0.982
Stroke (ischemic or hemorrhagic)	3.564 (2.468–5.146)	**<0.001**	3.182 (2.216–4.569)	**<0.001**	2.359 (1.643–3.389)	**<0.001**	2.451 (1.694–3.544)	**<0.001**	2.519 (1.619–3.920)	**<0.001**	2.220 (1.426–3.455)	**<0.001**	1.738 (1.117–2.706)	**0.014**	1.730 (1.110–2.696)	**0.015**
Gastro-intestinal bleeding	4.820 (3.447–6.740)	**<0.001**	3.754 (2.682–5.253)	**<0.001**	2.519 (1.799–3.525)	**<0.001**	2.687 (1.916–3.770)	**<0.001**	4.619 (3.232–6.600)	**<0.001**	3.910 (2.735–5.590)	**<0.001**	2.732 (1.910–3.907)	**<0.001**	2.760 (1.926–3.954)	**<0.001**
Transfusion of blood constituents	3.445 (3.104–3.823)	**<0.001**	2.984 (2.687–3.314)	**<0.001**	1.935 (1.740–2.151)	**<0.001**	2.317 (2.077–2.584)	**<0.001**	2.349 (2.090–2.640)	**<0.001**	2.133 (1.897–2.399)	**<0.001**	1.487 (1.321–1.674)	**<0.001**	1.648 (1.461–1.858)	**<0.001**
**Treatments**																
Amputation	2.737 (2.469–3.035)	**<0.001**	2.270 (2.045–2.520)	**<0.001**	0.976 (0.875–1.089)	0.665	1.830 (1.643–2.040)	**<0.001**	2.326 (2.054–2.633)	**<0.001**	1.954 (1.723–2.216)	**<0.001**	0.947 (0.832–1.078)	0.412	1.619 (1.424–1.840)	**<0.001**
Minor amputation	1.830 (1.606–2.084)	**<0.001**	1.507 (1.322–1.717)	**<0.001**	0.668 (0.585–0.763)	**<0.001**	1.202 (1.051–1.375)	**0.007**	1.528 (1.281–1.822)	**<0.001**	1.304 (1.093–1.556)	**0.003**	0.642 (0.537–0.768)	**<0.001**	1.074 (0.898–1.284)	0.436
Major amputation	4.437 (3.924–5.017)	**<0.001**	3.777 (3.338–4.273)	**<0.001**	1.927 (1.699–2.187)	**<0.001**	3.018 (2.659–3.426)	**<0.001**	3.269 (2.827–3.780)	**<0.001**	2.732 (2.359–3.163)	**<0.001**	1.501 (1.293–1.741)	**<0.001**	2.293 (1.975–2.661)	**<0.001**
Peripheral endovascular intervention of the lower extremity	0.598 (0.521–0.686)	**<0.001**	0.506 (0.441–0.581)	**<0.001**	0.402 (0.350–0.462)	**<0.001**	0.496 (0.432–0.571)	**<0.001**	0.511 (0.436–0.599)	**<0.001**	0.456 (0.389–0.535)	**<0.001**	0.397 (0.339–0.466)	**<0.001**	0.459 (0.391–0.538)	**<0.001**
Surgical peripheral artery revascularization of the lower extremity	0.782 (0.692–0.884)	**<0.001**	0.852 (0.754–0.963)	**0.010**	0.786 (0.696–0.888)	**<0.001**	0.830 (0.734–0.938)	**0.003**	0.744 (0.642–0.863)	**<0.001**	0.806 (0.695–0.934)	**0.004**	0.738 (0.636–0.856)	**<0.001**	0.763 (0.658–0.885)	**<0.001**
Intensive care unit	3.154 (2.759–3.605)	**<0.001**	3.081 (2.698–3.519	**<0.001**	2.232 (1.953–2.551)	**<0.001**	2.196 (1.912–2.523)	**<0.001**	2.455 (2.084–2.892)	**<0.001**	2.505 (2.126–2.951)	**<0.001**	1.865 (1.581–2.199)	**<0.001**	1.699 (1.435–2.011)	**<0.001**

* Adjustment: Adjusted for age, obesity, hyperlipidemia, cancer, coronary artery disease, heart failure, chronic obstructive pulmonary disease, essential arterial hypertension, acute and/or chronic kidney failure, diabetes mellitus and atrial fibrillation/flutter.

**Table 3:** Impact of female sex vs. male sex on in-hospital case fatality and adverse events during in-hospital stay in patients admitted due to PAD of any age (univariate and multivariate logistic regression model). Bold indicates significant differences at *p* ≤ 0.05.

	Univariate Regression Model		Multivariate Regression Model *		Multivariate Regression Model Adjusted for Age and Fontaine Classification		Multivariate Regression Model **	
	OR (95% CI)	*p*-Value	OR (95% CI)	*p*-Value				
**Adverse events during in-hospital stay**								
In-hospital case-fatality	1.385 (1.070–1.793)	**0.013**	1.287 (0.978–1.694)	0.071	1.245 (0.953–1.626)	0.108	1.294 (0.983–1.704)	0.066
MACCE	1.219 (0.970–1.531)	0.089	1.204 (0.944–1.535)	0.134	1.118 (0.883–1.415)	0.354	1.209 (0.948–1.543)	0.126
In-hospital stay > 10 days	1.053 (0.891–1.244)	0.545	1.007 (0.844–1.201)	0.940	0.976 (0.819–1.163)	0.784	0.993 (0.829–1.189)	0.936
In-hospital stay > 20 days			0.966 (0.835–1.118)	0.642	0.943 (0.816–1.088)	0.419	0.958 (0.824–1.113)	0.576
Pneumonia	0.753 (0.578–0.982)	**0.036**	0.710 (0.537–0.939)	**0.017**	0.724 (0.551–0.952)	**0.021**	0.706 (0.533–0.935)	**0.015**
Deep venous thrombosis and/or thrombophlebitis	1.149 (0.650–2.033)	0.632	1.210 (0.669–2.186)	0.528	1.206 (0.670–2.170)	0.533	1.206 (0.667–2.181)	0.536
Pulmonary embolism	1.248 (0.467–3.334)	0.658	1.401 (0.508–3.865)	0.515	1.490 (0.546–4.067)	0.437	1.405 (0.507–3.896)	0.513
Acute renal failure	0.999 (0.784–1.273)	0.993	1.001 (0.764–1.312)	0.995	0.999 (0.778–1.282)	0.992	0.977 (0.744–1.285)	0.870
Cardio-pulmonary resuscitation	0.893 (0.547–1.458)	0.651	1.088 (0.652–1.816)	0.747	1.039 (0.628–1.718)	0.861	1.082 (0.646–1.813)	0.764
Myocardial infarction	0.829 (0.481–1.427)	0.498	1.090 (0.613–1.938)	0.768	0.832 (0.475–1.457)	0.519	1.129 (0.632–2.015)	0.682
Stroke (ischemic or hemorrhagic)	0.829 (0.469–1.466)	0.519	0.803 (0.442–1.456)	0.469	0.767 (0.427–1.380)	0.377	0.809 (0.446–1.469)	0.487
Gastro-intestinal bleeding	1.107 (0.679–1.803)	0.685	1.055 (0.633–1.757)	0.838	1.042 (0.630–1.725)	0.871	1.062 (0.637–1.771)	0.818
Transfusion of blood constituents	0.966 (0.826–1.129)	0.665	1.112 (0.940–1.314)	0.215	1.067 (0.907–1.255)	0.435	1.121 (0.937–1.341)	0.213
**Treatments**								
Amputation	0.738 (0.628–0.867)	**<0.001**	0.779 (0.657–0.923)	**0.004**	0.782 (0.659–0.927)	**0.005**	0.771 (0.646–0.919)	**0.004**
Minor amputation	0.597 (0.480–0.743)	**<0.001**	0.611 (0.486–0.767)	**<0.001**	0.624 (0.498–0.783)	**<0.001**	0.599 (0.472–0.759)	**<0.001**
Major amputation	0.840 (0.695–1.016)	0.072	0.927 (0.760–1.131)	0.455	0.913 (0.749–1.113)	0.368	0.925 (0.756–1.132)	0.449
Peripheral endovascular intervention of the lower extremity	0.916 (0.742–1.130)	0.411	0.924 (0.742–1.150)	0.479	0.924 (0.744–1.148)	0.477	0.922 (0.740–1.148)	0.468
Surgical peripheral artery revascularization of the lower extremity	0.785 (0.648–0.951)	**0.013**	1.013 (0.827–1.241)	0.903	0.988 (0.809–1.207)	0.904	1.015 (0.828–1.244)	0.886
Intensive care unit	0.764 (0.619–0.944)	**0.012**	0.984 (0.784–1.234)	0.889	0.934 (0.751–1.163)	0.542	0.965 (0.757–1.229)	0.779

* Adjustment: Adjusted for age, obesity, hyperlipidemia, cancer, coronary artery disease, heart failure, chronic obstructive pulmonary disease, essential arterial hypertension, acute and/or chronic kidney failure, diabetes mellitus and atrial fibrillation/flutter. ** Adjustment: Adjusted for obesity, hyperlipidemia, cancer, coronary artery disease, heart failure, chronic obstructive pulmonary disease, essential arterial hypertension, acute and/or chronic kidney failure, diabetes mellitus, atrial fibrillation/flutter, surgical peripheral artery revascularization of the lower extremity and peripheral endovascular intervention of the lower extremity.

## Data Availability

All codes used in this study are publicly available online. The data/results are available at the Federal Statistical Office of Germany (Statistisches Bundesamt, DEStatis) (source: RDC of the Federal Statistical Office and the Statistical Offices of the federal states, DRG Statistics 2005–2020, and own calculations).

## Supplementary Materials

The following supporting information can be downloaded at: https://www.mdpi.com/article/10.3390/muscles5030063/s1, Figure S1: Directed acyclic graph (DAG) for different parameters and their impact on in-hospital death in PAD patients with sarcopenia [50]; Table S1: Age-dependent impact of female sex on in-hospital case-fatality rate in sarcopenic PAD patients; Table S2: Age-dependent impact of female sex on MACCE rate in sarcopenic PAD patients; Table S3: Age-dependent impact of female sex on prolonged length of in-hospital stay >20 days in sarcopenic PAD patients.
