# Supplementary material for: Sex-Specific Differences in Patients with Sarcopenia and Peripheral Artery Disease
Source: Muscles. 2026 Sep 14;5(3):63. doi: 10.3390/muscles5030063 (PMC13610119; doi:10.3390/muscles5030063)
Supplement: Supplementary file 1 [file muscles-05-00063-s001.zip › muscles-4433765-supplementary.pdf]

Supplementary material

Sex-specific differences in patients with sarcopenia and peripheral artery disease

Volker H. Schmitt, MD[1]; Omar Hahad, PhD[1,2]; Christoph Brochhausen, MD[4]; Thomas Münzel, MD[1,2]; Philipp Lurz, MD[1,2]; Christine Espinola-Klein, MD[1,3]; Lukas Hobohm, MD[1,3]; Karsten Keller, MD[1,3]

[1] Department of Cardiology, University Medical Center of the Johannes Gutenberg-University of Mainz, Germany

[2] German Center for Cardiovascular Research (DZHK), Partner Site Rhine Main, Mainz, Germany

[3] Center for Thrombosis and Hemostasis (CTH), University Medical Center of the Johannes Gutenberg-University of Mainz, Germany

[4] Institute of Pathology, Medical Faculty Mannheim, University Heidelberg, Germany

Short title: Sex-differences in sarcopenic patients with peripheral artery disease

Figure S1: Directed acyclic graph (DAG) for different parameters and their impact on in-hospital death in PAD patients with sarcopenia (Johannes Textor, Maciej Liśkiewicz: Adjustment Criteria in Causal Diagrams: An Algorithmic Perspective. In Proceedings of the 27th Conference on Uncertainty in Artificial Intelligence (UAI 2011), pp. 681-688. AUAI press, 2011.)

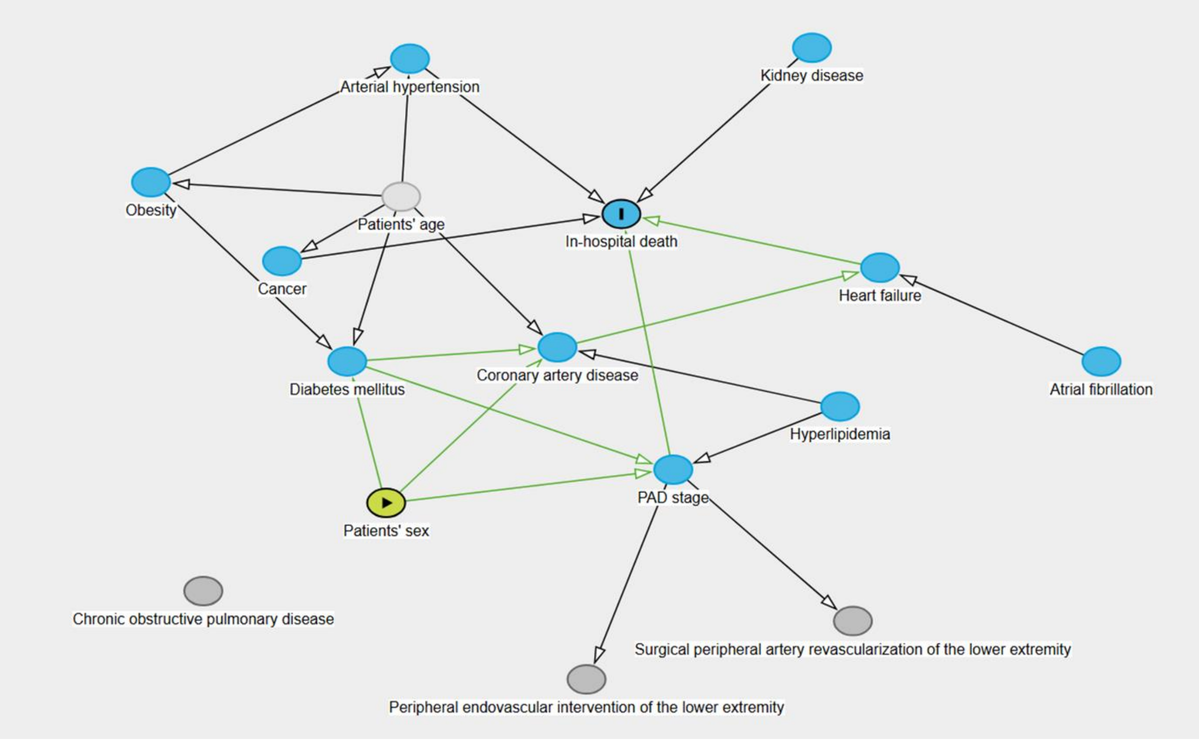

Table S1: Age-dependent impact of female sex on in-hospital case-fatality rate in sarcopenic PAD patients

Age decade	Univariate logistic regression		Multivariate logistic regression with adjustment I		Multivariate logistic regression with adjustment II		Multivariate logistic regression with adjustment III	
	**OR (95%CI)**	**P-value**	**OR (95%CI)**	**P-value**	**OR (95%CI)**	**P-value**	**OR (95%CI)**	**P-value**
**50 – 59 years**	1.045 (0.204-5.367)	0.958	0.905 (0.172-4.754)	0.906	7.440 (0.572-96.833)	0.125	5.105 (0.288-90.343)	0.266
**60 – 69 years**	1.470 (0.878-2.463)	0.143	1.594 (0.943-2.694)	0.082	1.663 (0.962-2.874)	0.069	1.683 (0.971-2.918)	0.064
**70 – 79 years**	1.017 (0.558-1.853)	0.956	1.110 (0.600-2.056)	0.739	1.244 (0.660-2.347)	0.499	1.249 (0.659-2.368)	0.496
**80 – 89 years**	1.005 (0.546-1.850)	0.988	1.037 (0.559-1.923)	0.909	1.036 (0.551-1.949)	0.913	1.009 (0.531-1.916)	0.979
**≥ 90 years**	1.595 (0.687-3.705)	0.278	1.882 (0.798-4.437)	0.148	1.523 (0.627-3.699)	0.352	1.737 (0.698-4.320)	0.235

Adjustment I: adjustment for PAD stage according Fontaine classification and Charlson Index

Adjustment II: adjustment for obesity, hyperlipidemia, cancer, coronary artery disease, heart failure, chronic obstructive pulmonary disease, essential arterial hypertension, acute and/or chronic kidney failure, diabetes mellitus and atrial fibrillation/flutter

Adjustment III: adjustment for obesity, hyperlipidemia, cancer, coronary artery disease, heart failure, chronic obstructive pulmonary disease, essential arterial hypertension, acute and/or chronic kidney failure, diabetes mellitus, atrial fibrillation/flutter, surgical peripheral artery revascularization of the lower extremity and peripheral endovascular intervention of the lower extremity

Table S2: Age-dependent impact of female sex on MACCE rate in sarcopenic PAD patients

**Age decade**	**Univariate logistic regression**		**Multivariate logistic regression with adjustment I**		**Multivariate logistic regression with adjustment II**		**Multivariate logistic regression with adjustment III**	
	**OR (95%CI)**	**P-value**	**OR (95%CI)**	**P-value**	**OR (95%CI)**	**P-value**	**OR (95%CI)**	**P-value**
**50 – 59 years**	1.605 (0.385-6.689)	0.516	1.373 (0.320-5.889)	0.669	5.990 (0.845-42.441)	0.073	6.774 (0.829-55.380)	0.074
**60 – 69 years**	1.425 (0.899-2.258)	0.131	1.579 (0.985-2.531)	0.058	1.625 (1.002-2.635)	**0.049**	1.634 (1.006-2.655)	**0.047**
**70 – 79 years**	1.055 (0.622-1.788)	0.842	1.153 (0.670-1.983)	0.608	1.282 (0.736-2.234)	0.381	1.271 (0.726-2.225)	0.401
**80 – 89 years**	0.907 (0.529-1.557)	0.723	0.932 (0.539-1.613)	0.802	0.967 (0.551-1.697)	0.908	0.929 (0.526-1.642)	0.801
**≥ 90 years**	1.672 (0.722-3.869)	0.230	1.982 (0.844-4.658)	0.116	1.594 (0.660-3.846)	0.300	1.829 (0.740-4.522)	0.191

Adjustment I: adjustment for PAD stage according Fontaine classification and Charlson Index

Adjustment II: adjustment for obesity, hyperlipidemia, cancer, coronary artery disease, heart failure, chronic obstructive pulmonary disease, essential arterial hypertension, acute and/or chronic kidney failure, diabetes mellitus and atrial fibrillation/flutter

Adjustment III: adjustment for obesity, hyperlipidemia, cancer, coronary artery disease, heart failure, chronic obstructive pulmonary disease, essential arterial hypertension, acute and/or chronic kidney failure, diabetes mellitus, atrial fibrillation/flutter, surgical peripheral artery revascularization of the lower extremity and peripheral endovascular intervention of the lower extremity

Table S3: Age-dependent impact of female sex on prolonged length of in-hospital stay >20 days in sarcopenic PAD patients

Age decade	Univariate logistic regression		Multivariate logistic regression with adjustment I		Multivariate logistic regression with adjustment II		Multivariate logistic regression with adjustment III	
	**OR (95%CI)**	**P-value**	**OR (95%CI)**	**P-value**	**OR (95%CI)**	**P-value**	**OR (95%CI)**	**P-value**
**50 – 59 years**	0.905 (0.462-1.773)	0.771	0.691 (0.328-1.453)	0.329	0.675 (0.300-1.517)	0.341	0.606 (0.251-1.460)	0.264
**60 – 69 years**	1.243 (0.916-1.687)	0.162	1.309 (0.957-1.791)	0.092	1.312 (0.959-1.795)	0.090	1.294 (0.938-1.785)	0.116
**70 – 79 years**	0.958 (0.703-1.307)	0.788	0.969 (0.705-1.331)	0.844	0.980 (0.712-1.348)	0.899	0.968 (0.697-1.343)	0.845
**80 – 89 years**	0.721 (0.509-1.023)	0.067	0.726 (0.511-1.030)	0.073	0.718 (0.500-1.031)	0.073	0.735 (0.509-1.061)	0.100
**≥ 90 years**	0.652 (0.389-1.093)	0.105	0.676 (0.401-1.140)	0.142	0.680 (0.389-1.187)	0.175	0.687 (0.386-1.221)	0.200

Adjustment I: adjustment for PAD stage according Fontaine classification and Charlson Index

Adjustment II: adjustment for obesity, hyperlipidemia, cancer, coronary artery disease, heart failure, chronic obstructive pulmonary disease, essential arterial hypertension, acute and/or chronic kidney failure, diabetes mellitus and atrial fibrillation/flutter

Adjustment III: adjustment for obesity, hyperlipidemia, cancer, coronary artery disease, heart failure, chronic obstructive pulmonary disease, essential arterial hypertension, acute and/or chronic kidney failure, diabetes mellitus, atrial fibrillation/flutter, surgical peripheral artery revascularization of the lower extremity and peripheral endovascular intervention of the lower extremity
